# Evaluation of the viability of microencapsulated *Trichoderma longibrachiatum* conidia as a strategy to prolong the shelf life of the fungus as a biological control agent

**DOI:** 10.3389/fchem.2024.1473217

**Published:** 2025-01-15

**Authors:** Luis Diego Arias-Chavarría, Diego Batista-Menezes, Steffany Orozco-Cayasso, Alejandro Vargas-Martínez, José Roberto Vega-Baudrit, Gabriela Montes de Oca-Vásquez

**Affiliations:** ^1^ Escuela de Ciencias Agrarias, Universidad Nacional, Heredia, Costa Rica; ^2^ National Nanotechnology Laboratory, National Center for High Technology, San José, Costa Rica; ^3^ Laboratorio de Fitopatología, Escuela de Ciencias Agrarias, Universidad Nacional, Heredia, Costa Rica; ^4^ Center for Sustainable Development Studies, Universidad Técnica Nacional, Alajuela, Costa Rica

**Keywords:** microcapsules, alginate, nanocellulose, chitosan, phytopathogenic controller

## Abstract

*Trichoderma* is an antagonistic fungus used commercially; however, the viability of these formulations is affected by biotic and abiotic factors. In this research, microcapsules of sodium alginate reinforced with nanocellulose and/or chitosan were developed to encapsulate *T. longibrachiatum* conidia and characterized by SEM, FTIR, and TGA. The viability of the microencapsulated conidia was evaluated through different temperatures (room temperature, 5°C and 37°C), as well as their *in vitro* antagonistic potential against *Fusarium oxysporum*. The formulations evaluated had encapsulation efficiencies above 92% and the microcapsules with alginate, chitosan, and nanocellulose maintained 100% viability at 37°C for 2 months. In addition, all formulations evaluated retained antagonistic ability against *F. oxysporum*. These findings support the use of alginate, nanocellulose and chitosan for the formulation of microcapsules to maintain the viability of *T. longibrachiatum* conidia over time and at different temperature conditions.

## 1 Introduction

The continuous use of agrochemicals for the management of plant diseases has generated several environmental and health problems, so biological control strategies have gained importance in recent years (Chandrika et al., 2019; Sehrawat et al., 2022). The use of beneficial microorganisms such as fungi and bacteria (Herrera et al., 2020; Asaturova et al., 2021; Elnahal et al., 2022), has an advantage that it allows the control of pests and diseases, and reduces the risk of pesticide resistance development (Saberi-Riseh et al., 2021). Fungi of the genus *Trichoderma* are used as biological control agents (BCAs) and plant growth promoters (PGPFs) (Saldaña-Mendoza et al., 2023; Martinez et al., 2023). *T. longibrachiatum* species have been evaluated in disease and pest control, the production of metabolites involved in these processes, as well as the compounds it stimulates in plants under stress conditions (Sornakili et al., 2020; Sridharan et al., 2021; TariqJaveed et al., 2021; Poveda, 2021).

Several strategies have been developed to create *Trichoderma* formulations from the fungal biomass (Martinez et al., 2023), however, these products have certain disadvantages, such as low viability and stability (Løvschall et al., 2024), which makes it difficult for these microorganisms to establish themselves and express their full antagonistic potential for the control of phytopathogens or pests (Braga et al., 2019). Formulation development must ensure the protection and maintenance of the viability of conidia or mycelium of antagonistic fungi (Mulatu et al., 2021; Saldaña-Mendoza et al., 2023). Several strategies have been employed for the preservation of *Trichoderma* spp. conidia. These include the use of solid carriers, such as vermiculite and biochar, liquid carriers like oils and hydrogels, and processing techniques such as freeze-drying, spray-drying, and fluidized bed drying (Martinez et al., 2023). In a study conducted by Kaewkhon et al. (2024), *T. longibrachiatum* conidia were preserved by developing pellets using spent mushroom substrate. This approach was shown to extend conidial shelf life and enhance their efficiency for biological control.

Moreover, encapsulation of conidia is a strategy that can be used to overcome environmental constraints and give the antagonist a competitive advantage over pathogens and another microflora (Locatelli et al., 2018; Saberi-Riseh et al., 2021). Microcapsules are usually produced from the formation of liquid droplets, either by dripping or by emulsifying and solidifying the liquid droplets to form particles (Bejarano and Puopolo, 2020). Various polymers have been used for the encapsulation of active compounds (Martínez et al., 2023; Lotfalinezhad et al., 2024) and biological control agents, among which are alginate, starch, pectin, cellulose, carrageenan, agar, and chitosan (Chaudhary and Shukla, 2020; Saberi-Riseh et al., 2021).

Alginate is a natural anionic polysaccharide derived from brown algae, composed of residues of β-d-mannuronate and α-l-guluronate, linked by 1,4 glycosidic bonds (Murugappan and Muthadhi, 2022; Cao et al., 2023). This has been used to microencapsulate *Trichoderma* conidia to maintain the viability of formulations (Locatelli et al., 2018; Mancera et al., 2019; Adzmi et al., 2021), as well as enhance their antagonistic characteristics (Løvschall et al.2024), as it has been found to exhibit synergism with *Trichoderma* for disease control (Singh and Chittenden, 2021). Moreover, chitosan has been used in agriculture in the encapsulation of agrochemicals and micronutrients (García et al., 2023). Otherwise, nanocellulose, obtained from plant cellulose or synthesized by some bacteria, has been utilized in the formulation of micro- and nanoparticles, owing to its ability to enhance the mechanical and physical properties of these materials (Gan et al., 2020).

To date, very little is known about the use of additives such as chitosan and nanocellulose to improve the characteristics of alginate microcapsules, particularly their mechanical and physical properties (Siqueira et al., 2019). Chitosan, in particular, helps enhance these properties by increasing the rigidity of the microcapsules (Qu and Luo, 2020). Some studies have shown that encapsulating *Trichoderma* spp. conidia in alginate microcapsules, compared to non-encapsulated conidia, significantly improves their viability and stability in field trials under various environmental stresses, such as heat and UV treatments, while also extending their activity during storage at different temperatures (Maruyama, et al., 2020; Qi et al., 2023). However, studies are lacking to understand how factors such as storage time, temperature, and humidity affect the viability of fungal formulations, mainly under more realistic conditions of *Trichoderma* exposure in the field (Locatelli et al., 2018). Due to the above, in the present study, the effect of microencapsulation of *T. longibrachiatum* was evaluated by measuring the viability of conidia when exposed to different temperature conditions, as a strategy to prolong the shelf life of the fungus as a biological control agent, founding that the microcapsules with alginate, chitosan, and nanocellulose maintained 100% viability at 37°C for 2 months. We hypothesize that adding chitosan and nanocellulose to the microcapsule formulation allows a better protection of the conidia while preserving their viability in adverse conditions.

## 2 Materials and methods

### 2.1 Microorganism

The fungus *T. longibrachiatum* was provided by the Phytopathology Laboratory of the School of Agricultural Sciences of the Universidad Nacional of Costa Rica, which had been previously recovered from the rhizosphere of soils destined to pineapple (*Ananas comosus* L. Merr) production in Puerto Viejo, Sarapiquí, with the purpose of carrying out tests for the control of *Fusarium oxysporum*, the second most important phytopathogen in this crop in Costa Rica. *T. longibrachiatum* was under storage conditions at 10°C in vials with sterile mineral oil.

### 2.2 Reactivation of *Trichoderma longibrachiatum*

The isolates stored in vials were transferred to Petri dishes containing potato-dextrose-agar (PDA) culture medium, which were incubated for 7 days at 28°C, until the colonies were densely populated and sporulated covering the dishes. Subsequently, for further recovery of the growth vigor of the isolates, 1 cm diameter discs were cut in the region between the center and the edge of the colonies and transferred to new dishes containing PDA culture medium. These were incubated for 7 days at room temperature.

### 2.3 Spore production of *T.* longibrachiatum

A plate with pure and sporulated culture of the *T. longibrachiatum* strain was taken, the mycelium was scraped with a sterile scalpel and placed in 20 mL of sterile distilled water. The concentration of the conidial suspension was determined with the aid of a hemocytometer, according to the methodology described by French and Hebert (1980). For this, a Neubauer chamber was used and a count of the number of propagules in five secondary squares was performed. Finally, the suspensions were adjusted to obtain a concentration (1 × 10^6^ conidia mL^−1^) and to be able to use them for microencapsulation and conventional formulation.

### 2.4 Conventional formulation of *Trichoderma longibrachiatum*

In the conventional formulation (TL) of *T. longibrachiatum*, 100 g of rice was placed in a beaker and washed with water until the water was not cloudy. Subsequently, the antibiotic chloramphenicol was added, the mixture was shaken, and left to stand for 5 min. The water with the antibiotic was discarded and the rice was placed in hermetically sealed polystyrene bags. The rice was autoclaved for 40 min at 121°C, with 1.2 kg/cm^2^. Once sterilized, an aliquot of 5 mL of the conidia suspension (1 × 10^6^ conidia mL^−1^), described above, was placed in the rice contained in the bag, sealed and incubated at room temperature until mycelial growth and spore production were observed. Once the substrate with the inoculum was ready, 25 g were placed in plastic vials for viability tests. This test was conducted by placing 50 mg of the TL in plastic containers, exposed to storage at different temperature conditions: room temperature (20°C), 5°C and 37°C with a relative humidity of 80% in bioclimatic chambers. Conidial viability assessments were performed at 3, 5, 7, 15, 30, and 60 days of exposure and viability was determined by direct counts of viable and non-viable conidia after 24 h, using an optical microscope at ×40 magnification.

### 2.5 Preparation of nanocellulose

A 1.2% solution of cotton cellulose (fibers (medium), Sigma-Aldrich, MO, United States) was prepared and placed in an ultrasonic bath for 10 min. Subsequently, the solution was sonicated for 1 hour at 700 W (QSONICA Q700, Newton, CT, United States), with an amplitude of 40% and cycles of 50 s ON and 10 s OFF, during the sonication the sample was kept cold. Subsequently, the solution was allowed to stand for 30 min to precipitate and the supernatant was extracted, which was centrifuged at 3,500 rpm for 5 min. The precipitate obtained was discarded and the supernatant was centrifuged twice at 10,000 rpm for 5 min, preserving the precipitate in both centrifugations. Finally, the precipitate was dried in the oven at 35°C and stored at 4°C for further SEM analysis.

### 2.6 Experimental design of microcapsules formulation

Microcapsules were developed following the methodology described by Lupo et al. (2014) with some modifications. To standardize the method, the type of surfactant (Span 80 and Tween 20) was first evaluated at 5% and then the concentration of the surfactant Tween 20 (5%, 7.5%, and 10%). Once the type and concentration of surfactant were determined, different agitation speeds were compared (600, 800 and 1,000 rpm), and with the best result obtained, three concentrations of chitosan (Acros Organics, CAS:9012-76-4) and nanocellulose (0.25%, 0.5%, and 1%) were evaluated as coating and reinforcement polymers, respectively.

For the formulation with alginate (Alg), a 10 mL solution of 2% (w/w) sodium alginate (Sigma-Aldrich, CAS:9005-38-3) was prepared to which 1 mL of a 0.5 M CaCO_3_ solution was added. Subsequently, it was mixed with a homogenizer (Ultra-Turrax, IKA, T25, Germany) for 3 min at 12,000 rpm and left under refrigeration for 1 h to hydrate and remove air bubbles. The above mixture (dispersed phase) was dispersed in 40 mL of soybean oil with 5% (w/w) Tween 20 (oil phase). To promote the formation of the water-in-oil (W/O) emulsion, the dispersed phase was delivered dropwise in a controlled manner using a syringe with constant agitation for 15 min at 1,000 rpm. Subsequently, gelation of the emulsion sodium alginate droplets was induced by the controlled addition of a mixture consisting of 10 mL soybean oil and 80 μL of glacial acetic acid. The microcapsules formed were separated from the oil phase with the addition of 100 mL of aqueous CaCl_2_ solution 0.05 M (Sigma-Aldrich, CAS:10043-52-4) containing 10% (w/w) Tween 20 under agitation for 15 min at 650 rpm.

For the formulations of alginate with chitosan (0.5%) (AlgCh), alginate and nanocellulose (0.5%) (AlgNc) and alginate, chitosan (0.5%) and nanocellulose (0.5%) (AlgChNc), the methodology described above was followed, with the addition of the polymers to the dispersed phase with the sodium alginate.

### 2.7 Microencapsulation of *Trichoderma longibrachiatum*

Microencapsulation of the fungal conidia was carried out following the methodology described in the previous point, with the addition of 10 mL of conidial suspension (1 × 10^6^ conidia ml^-1^) to the dispersed phase, followed by agitation for 20 min with the aid of a magnetic stirrer, before preparing the emulsion by mixing with soybean oil. The microcapsules obtained were filtered using a coffee filter and three washes were performed with sterile distilled water. Drying was carried out at room temperature for 24 h and the dried microcapsules were packaged for further analysis.

### 2.8 Characterization of microcapsules

#### 2.8.1 Fourier transform infrared spectroscopy (FTIR)

Microcapsules with and without conidia were analyzed in duplicate, using a Nicolet 6700 FT-IR infrared spectrometer (Thermo Fisher Scientific, United States). The samples were placed directly in the equipment without any pretreatment. A scan was performed in the range 500–4,000 cm^−1^. The spectra were analyzed with OMNIC 8.1 software (OMNIC Series 8.1.10, Thermo Fisher Scientific, United States).

#### 2.8.2 Thermogravimetric analysis (TGA)

Microcapsules without conidia were analyzed in duplicate using a TGA-Q500 thermogravimetric analyzer (TA Instruments, United States), equipped with Universal Analysis 2000 software (version 4.5A, TA Instruments, United States). A nitrogen purge flow rate of 40 mL/min and a sample purge flow rate of 60 mL/min were used. Initially, the equipment was kept at equilibrium at 25°C for 5 min and subsequently, heating was performed at 10°C/min, in a temperature range from 25°C to 600°C.

#### 2.8.3 Differential scanning calorimetry (DSC)

Microcapsules without conidia were analyzed in duplicate by differential scanning calorimetry (DSC) (Q200, TA Instruments, United States), using TA Universal Analysis software. Temperature ramps covering a cycle from 25°C to 200°C at a rate of 10°C/min were used for the analysis.

#### 2.8.4 Scanning electron microscopy (SEM)

Microcapsules with and without conidia were analyzed in duplicate, taking images at different magnifications, using SEM equipment (JEOL, JSM-6390 LV, Japan), operating at a voltage acceleration between 5 and 10 kV, with secondary electrons (SEI) and a spot size between 30 and 50.

### 2.9 Encapsulation efficiency

To determine the encapsulation efficiency, 50 mg of the microcapsules were added in 10 mL of a solution of NaHCO_3_ (0.2 M) and Na_2_C_6_H_5_O_7_·2H_2_O (0.06 M) and shaken for 2 h to achieve complete dissolution of the microcapsules. A 10 µL aliquot was extracted and conidia were counted using a Neubauer chamber under an optical microscope (Olympus Optical, CH, Japan). Encapsulation efficiency was defined from the amount of conidia added and the amount of conidia released from the dried microcapsules.

### 2.10 Conidial release rate

An optical microscope (Olympus Optical, CH, Japan) was used to determine the release rate of encapsulated conidia. The dried microcapsules (50 mg) were placed in a solution of NaHCO_3_ (0.2 M) and Na_2_C_6_H_5_O_7_·2H_2_O (0.06 M). The duration of the immersion was 0, 1, 5, 10, 30, 30, 60, 120, and 240 min (Liu and Liu, 2009). For each time, two samples were taken and analyzed under an optical microscope, determining the conidial concentration with the aid of a Neubauer chamber.

### 2.11 Conidial temperature tolerance

To determine the conidial tolerance of encapsulated and non-encapsulated conidia, 50 mg of each formulation were placed in plastic containers, which were exposed to storage at different temperature conditions: room temperature (20°C), 5°C and 37°C with a relative humidity of 80% in bioclimatic chambers. Conidial viability assessments were performed at 3, 5, 7, 15, 30, and 60 days of exposure and viability was determined by direct counts of viable and non-viable conidia after 24 h, using an optical microscope at ×40 magnification. Conidia were considered viable when the germ tube length was greater than the conidial diameter. For this, 5 mg of the microcapsules were taken and placed in a 2 mL tube to which 0.5 mL of a solution of NaHCO_3_ (0.2 M) and Na_2_C_6_H_5_O_7_·2H_2_O (0.06 M) were added. Vortex agitation was performed for 1 min to dissolve the microcapsules and a 25 μL aliquot was placed on a sterile slide containing a thin layer of Agar-Water medium on the upper side, the slide was contained inside a Petri dish with wet paper towel. The aliquot of microcapsules was spread with a glass rod over the medium and incubated for 24 h at room temperature. After incubation time, 20 conidia were counted randomly along the entire slide, counting germinated and non-germinated conidia.

### 2.12 Biological activity – *In vitro* antagonism

An *in vitro* antagonism test was performed to determine the antagonistic capacity of *T. longibrachiatum* after the microencapsulation process for the four formulations exposed at room temperature, 5°C and 37°C for 60 days. For this, the microcapsules were dissolved in a buffer (NaHCO_3_ (0.2 M) and Na_2_C_6_H_5_O_7_·2H_2_O (0.06 M)) and inoculated in Petri dishes containing PDA medium for growth. Once the fungus had sporulated (12 days), a 5 mm disc was taken, using a punch, and placed 1 cm from the edge of a Petri dish containing PDA medium, and at the other end, a 5 mm disc of the pathogen *F. oxysporum* 1 cm from the edge. The dish was incubated and evaluated at 5, 9, and 14 days to determine the antagonistic capacity of the microencapsulated fungus. This test was performed in duplicate for each of the formulations and the results were evaluated according to the Bell et al. (1982) class scale (Table 1).

**TABLE 1:** Class scale for the evaluation of the degree of *Trichoderma* antagonism.

Class	Description
1	*Trichoderma* completely overcame the pathogen and covered the entire surface of the medium
2	*Trichoderma* grew over at least two-thirds of the surface of the medium
3	*Trichoderma* and the pathogen each colonized approximately half the surface area of the medium (more than one-third and less than two-thirds) and neither organism appeared to dominate the other
4	The pathogen colonized at least two-thirds of the media surface and appeared to resist *Trichoderma* invasion
5	The pathogen completely outcompeted *Trichoderma* and occupied the entire surface of the medium

Source: Bell et al. (1982).

### 2.13 Experimental design

The experimental unit and the observational unit for the temperature and storage tests was a plastic container containing microcapsules for each formulation. The structure of the treatments for the tests was bifactorial, where the first factor corresponds to encapsulation and non-encapsulation, and the second factor was the temperature levels. In the case of storage, the encapsulation factor, and the time factor (days) were analyzed. Each treatment had three replicates and the structure of the trial was a completely randomized design (CRD).

### 2.14 Data analysis

For the variables evaluated in the research, the most important summary measures were estimated. For release rate and conidial tolerance to temperature, point and line plots were made by taking the means, using the ggplot2 package (Wickham, 2016) of the statistical software R version 4.1.2 (R Core Team, 2020).

To determine the mean encapsulation efficiency and release rate of microencapsulated conidia, a 95% confidence interval for the mean was estimated using the following formula:

$$L_{i}=\underline{x}\;\pm \;T_{1-\frac{\alpha }{2};n-1}\times \frac{s}{\sqrt{n}}$$

Where:

i = Lower limit and Upper limit

$\underline{x\;}$ = mean of the variable under study

$T_{1-\frac{\alpha }{2};n-1}$ = T-distribution statistic

s = standard deviation of the variable under study

n = sample size

To identify significant differences in particle size among the treatments without conidia (since the microparticles agglomerated, making it difficult to precisely determine particle size), the R software was used. The normality of the data was assessed using the Shapiro-Wilk test, and homoscedasticity was evaluated with Levene’s test. As the data did not follow a normal distribution, a Kruskal–Wallis H test was implemented to identify differences among the treatments (*p* < 0.05), followed by a Dunn’s *post hoc* test to determine pairwise differences between treatments.

## 3 Results

### 3.1 Standardization of microcapsule formulation

Tests were carried out to standardize the formulation of alginate microcapsules using the emulsion-internal gelation method. The surfactants Span 80, and Tween 20 were evaluated, which were used at 5% (w/w) in the oil phase of the emulsion and at 10% with CaCl_2_ for hardening and washing of the microcapsules. The oil phase enables the formation of the emulsion by incorporating sodium alginate (emulsion step), and the microcapsules are subsequently produced using a gelling agent (gelation step) (Lin et al., 2021).

**FIGURE 1:**
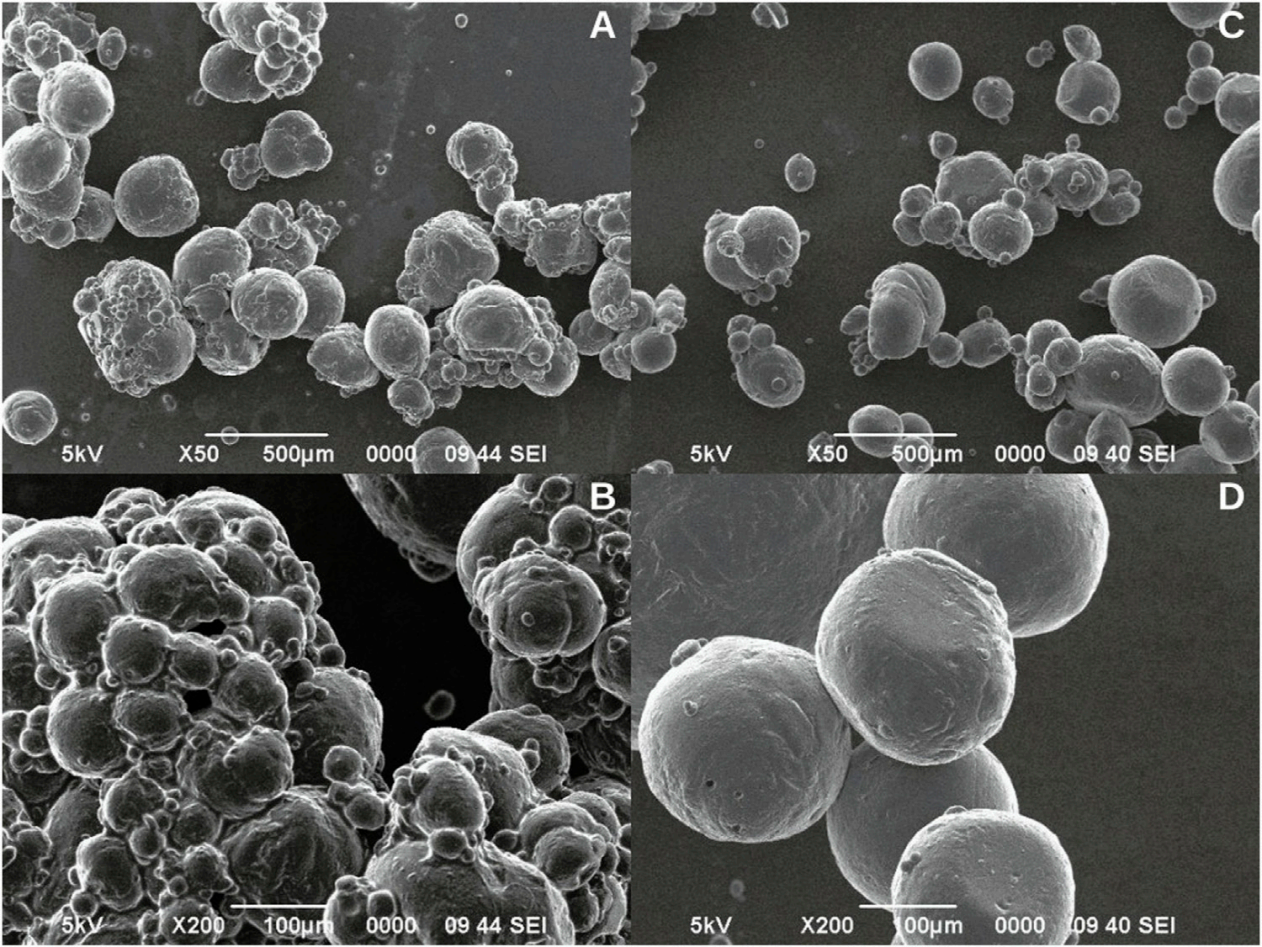
Microcapsules formulated with 5% Span 80 surfactant **(A, B)**. Microcapsules formulated with 5% Tween 20 surfactant **(C, D)**.

The microcapsules formulated using Span 80 had an average size of 261.84 ± 61.68 µm and showed regular circumference and slightly rough surface (Figure 1A). However, as seen in Figure 1B, they tended to agglomerate, and the observed sizes were very heterogeneous.

Otherwise, the microcapsules formulated with Tween 20 had an average size of 253.95 ± 96.85 µm and presented regular circumference, and smooth surface (Figure 1D). Moreover, the observed sizes were more homogeneous and no tendency to agglomeration was observed (Figure 1C).

**FIGURE 2:**
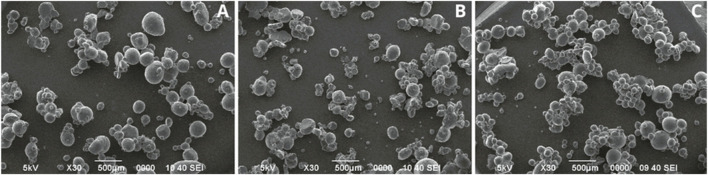
Microcapsules formulated at different agitation speeds with 5% Tween 20. **(A)** 600 rpm, **(B)** 800 rpm, **(C)** 1,000 rpm.

Once the surfactant was selected, the effect of agitation speed on the formation of the emulsion and the gelation reaction was analyzed, for which three speeds (600, 800 and 1,000 rpm) were evaluated (Figure 2). The microcapsules obtained at 600 rpm exhibited size diversity, with an average size of 286.67 ± 75.28 µm, a regular circumference, and a smooth surface (Figure 2A), while those formulated at 800 rpm presented greater size diversity, with an average size of 249.17 ± 99.57 µm, and irregular circumference (Figure 2B). The best result was obtained when an agitation speed of 1000 rpm was used, since these showed greater size homogeneity, with two sizes predominating (100 and 300 µm), with an average size of 190.83 ± 91.75 µm, as well as regular circumference and smooth surface (Figure 2C).

**FIGURE 3:**
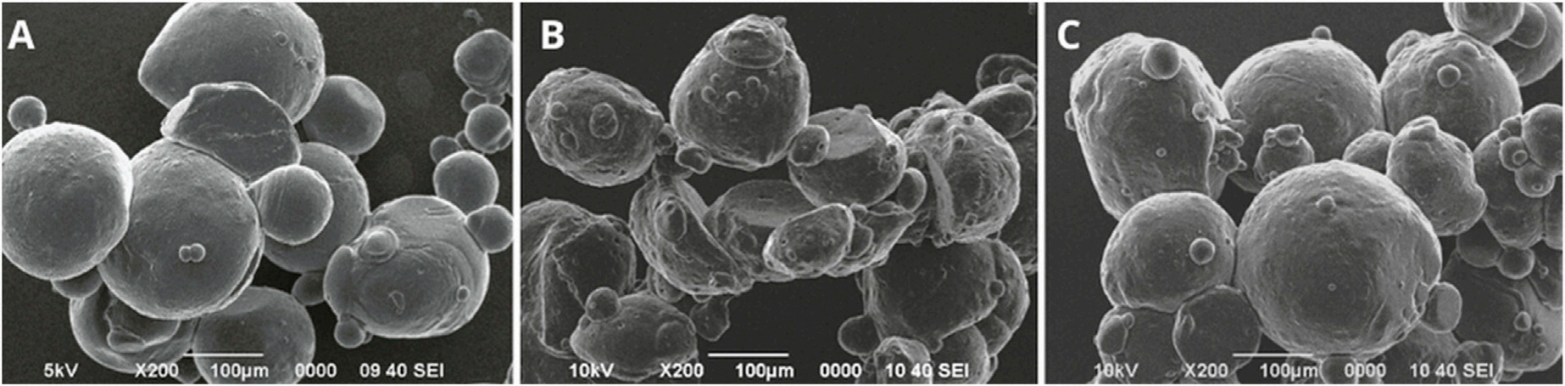
Evaluation of different concentrations of the surfactant Tween 20 at a stirring speed of 1,000 rpm. **(A)** 5%, **(B)** 7.5%, **(C)** 10%.

After identifying the agitation speed that presented the best results (1,000 rpm), the concentration of the surfactant Tween 20 (5%, 7.5%, and 10%) was evaluated (Figure 3). The microcapsules formulated at 7.5% had heterogeneity in size, with an average size of 100.99 ± 55.78 µm, irregular circumference, and rough surfaces (Figure 3B). Those formulated at 10% presented surfaces with less roughness than those formulated at 7.5%, with an average size of 136.91 ± 77.36 µm (Figure 3C). Microcapsules with Tween 20 at 5% presented regular circumference, smooth surface, and an average size of 124.81 ± 55.68 µm (Figure 3A).

### 3.2 Nanocellulose synthesis

**FIGURE 4:**
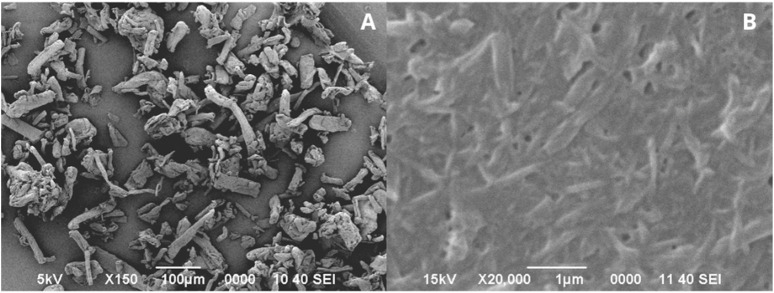
Cotton cellulose fibers **(A)**. Cotton nanocellulose fibers obtained from sonication treatment **(B)**.

The results of the synthesis of nanocellulose from cotton cellulose are shown in Figure 4. It is observed that after treatment with sonication the cellulose fibers with sizes between 10 and 50 µm (Figure 4A), were reduced, obtaining nanofiber sizes between 100 and 300 nm (Figure 4B), which were used in the preparation of microcapsules.

### 3.3 Characterization of microcapsules

#### 3.3.1 Scanning electron microscopy (SEM)

**FIGURE 5:**
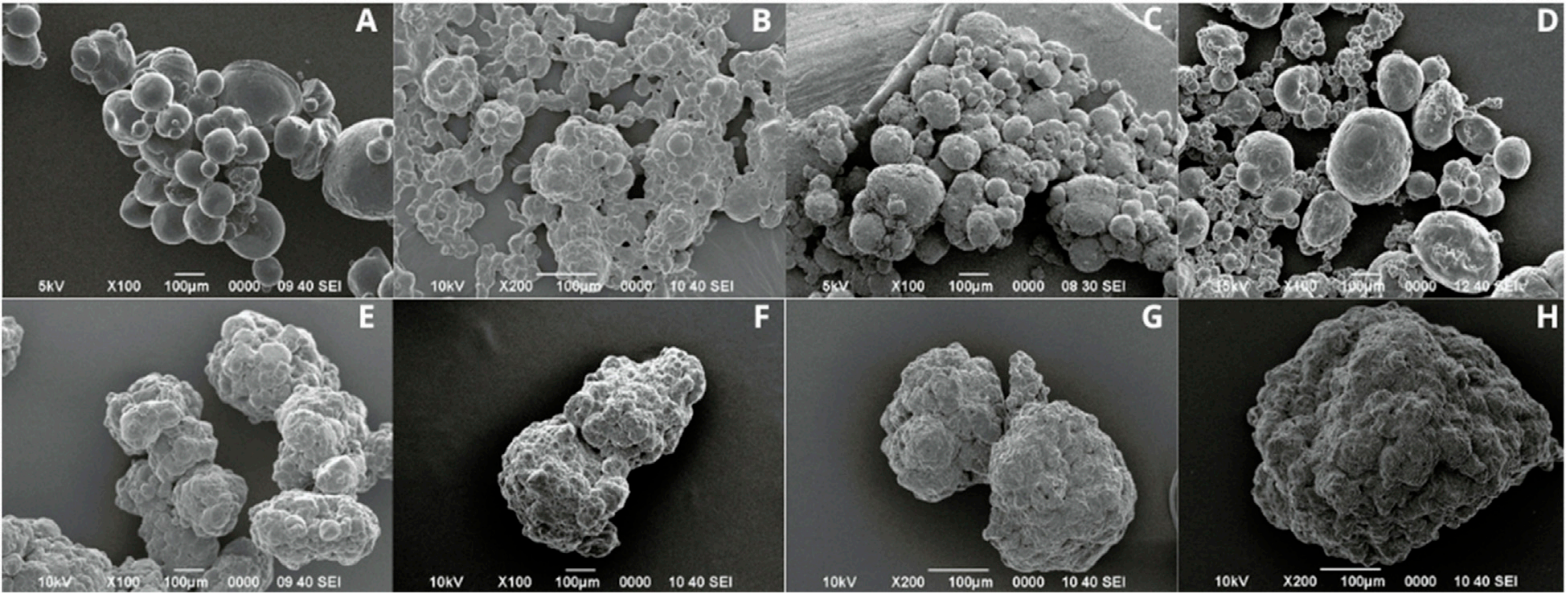
Microcapsules without conidia **(A–D)** and with conidia of *T. longibrachiatum* **(E–H)**. Microcapsules Alg **(A, E)**, AlgNc **(B, F)**, AlgCh **(C, G)**, AlgChNc **(D, H)**.

The results of the morphological characterization of the microcapsules with and without conidia are shown in Figure 5. The alginate microcapsules without conidia (Alg) had an average size of 153.67 ± 71.27 µm, with some smaller microcapsules around 50 µm. Additionally, a regular circumference and smooth surface were observed (Figure 5A). After the conidia encapsulation process, microcapsules smaller than 100 µm were observed; however, they were agglomerated, as shown in Figure 5E.

Otherwise, the alginate with nanocellulose formulation without conidia (AlgNc) produced microcapsules with the smallest size, averaging 29.67 ± 7.77 µm, with regular circumference and smooth surface, although agglomeration was observed (Figure 5B). As shown in Figure 5F, microcapsules with alginate, nanocellulose and conidia agglomerated and showed irregular circumference. For the alginate and chitosan (AlgCh) formulation, the microcapsules had an average size of 105.67 ± 30.63 µm, with a regular circumference and surface roughness (Figure 5C). Additionally, in the presence of conidia, the particles exhibited agglomeration (Figure 5G). Moreover, the microcapsules formulated with chitosan, alginate, and nanocellulose (AlgChNc) had an average size of 198.67 ± 64.27 µm. They exhibited a regular circumference and a surface with some roughness (Figure 5D). However, similar to the previous formulations, they tended to agglomerate and display rough surfaces after encapsulation (Figure 5H).

The Kruskal–Wallis H test revealed a significant difference in particle size among the different treatments without conidia (χ^2^ (3) = 24.68, p < .001). Post-hoc Dunn’s test with Bonferroni correction indicated that the average size of the AlgNc treatment was significantly smaller than that of the other treatments.

#### 3.3.2 Thermogravimetric analysis (TGA)

**FIGURE 6:**
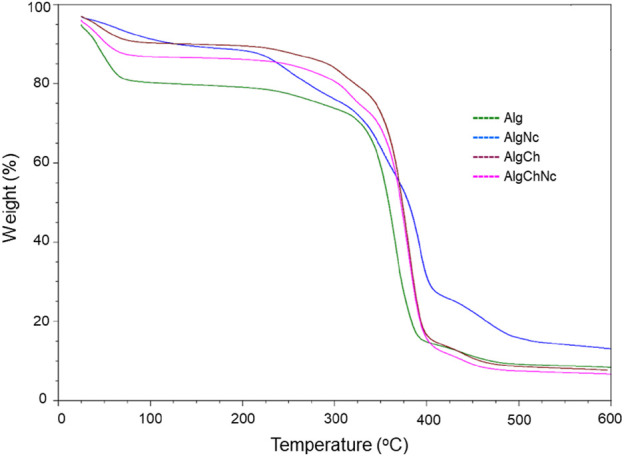
Thermogravimetric analysis (TGA) of microcapsules formulated with sodium alginate, chitosan and nanocellulose for the encapsulation of *T. longibrachiatum* conidia.

Figure 6 shows the thermogravimetric analysis of the formulated microcapsules without conidia. The results indicate that the microcapsules are relatively stable at temperatures below 220°C. The graph shows a first mass loss event between 25°C and 100°C. The second event occurs between 100°C and 350°C. Subsequently, a third event occurs from 350°C, with a mass loss of approximately 70% upon reaching 400°C, from which point the Alg, AlgCh and AlgChNc formulations remain constant. In the case of AlgNc, there is a mass loss of about 55% upon reaching 400°C, then another event with a 10% mass loss and remaining constant from 500°C onwards.

#### 3.3.3 Differential scanning calorimetry (DSC)

**FIGURE 7:**
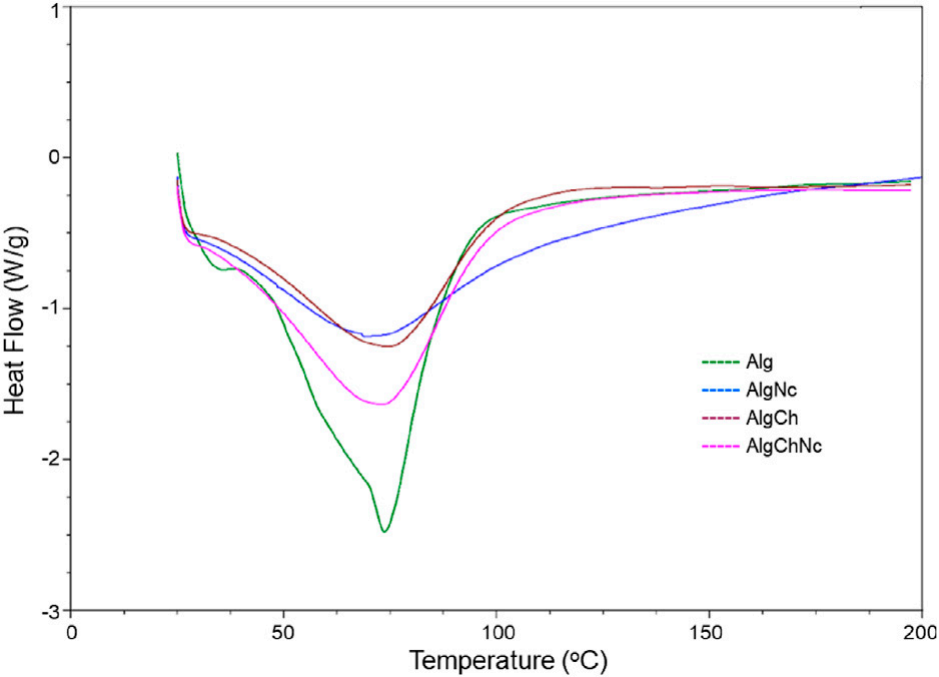
Differential Scanning Calorimetry (DSC) analysis of microcapsules formulated with sodium alginate, chitosan and nanocellulose for the encapsulation of *T. longibrachiatum* conidia.

The thermal transition curve of Alg, AlgNc, AlgCh and AlgChNc microcapsules was analyzed by DSC (Figure 7). The Alg formulation presented a first endothermic transition near 33°C and a subsequent endothermic peak at 75°C, also observed in AlgNc and AlgChNc microcapsules, whereas the AlgCh formulation showed a peak near 80°C.

#### 3.3.4 Fourier transform infrared spectroscopy (FTIR)

**FIGURE 8:**
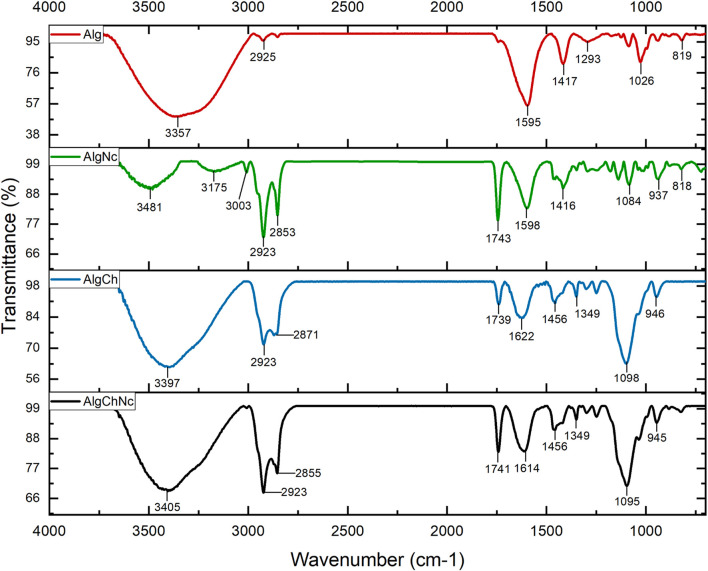
FTIR spectra of sodium alginate (Alg), sodium alginate and nanocellulose (AlgNc), sodium alginate and chitosan (AlgCh) and sodium alginate, chitosan and nanocellulose (AlgChNc) formulations.

**FIGURE 9:**
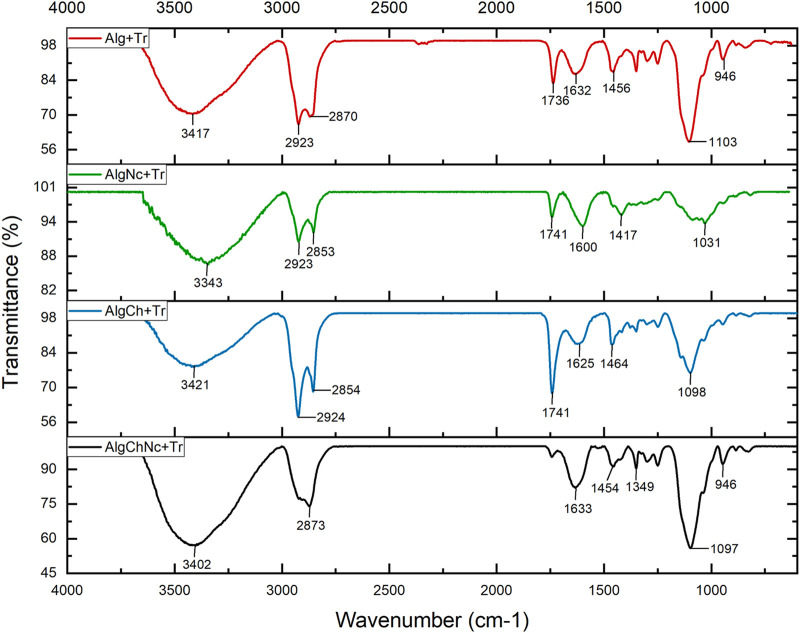
FTIR spectra of the microcapsule formulations with conidia of *T. longibrachiatum*. Sodium alginate (Alg + Tr), sodium alginate and nanocellulose (AlgNc + Tr), sodium alginate and chitosan (AlgCh + Tr) and sodium alginate, chitosan and nanocellulose (AlgChNc + Tr).

The functional groups and intermolecular interactions of the microcapsules and the reagents used were analyzed by FTIR. The absorption bands of sodium alginate, chitosan, nanocellulose, calcium chloride and calcium carbonate, reagents used in the different microcapsule formulations, are shown in (Supplementary Table S1). Figures 8, 9 shows the FTIR spectra of the microcapsule formulations without conidia and with conidia, respectively. It can be noted that both spectra are very similar.

Figures 8, 9 show the FTIR spectra of the microcapsule formulations without conidia and with conidia, respectively. It is evident that both spectra exhibit striking similarities. Notably, both spectra show a prominent absorption band at 3357 cm^−1^, along with distinct peaks observed at 1595 cm^−1^, 1417 cm^−1^ and 2,925 cm^−1^. Furthermore, there was a peak at 1622 cm^−1^ in the AlgCh microcapsules. In the AlgCh and AlgChNc formulations, the peak at 1592 cm^−1^ disappeared, and a new peak was observed at 1739 cm^−1^.

### 3.4 Encapsulation efficiency and conidial release rate

Table 2 shows the encapsulation efficiency of the conidia in the microcapsules. A high encapsulation efficiency of more than 92% was found in all formulations.

**TABLE 2:** Encapsulation efficiency of conidia in the different microcapsule formulations.

Formulation	Alg	AlgNc	AlgCh	AlgChNc
Encapsulation efficiency (%)	99 ± 9	92.5 ± 7.5	99 ± 2.5	96 ± 6

^*^Data represents the average ± standard error.

**FIGURE 10:**
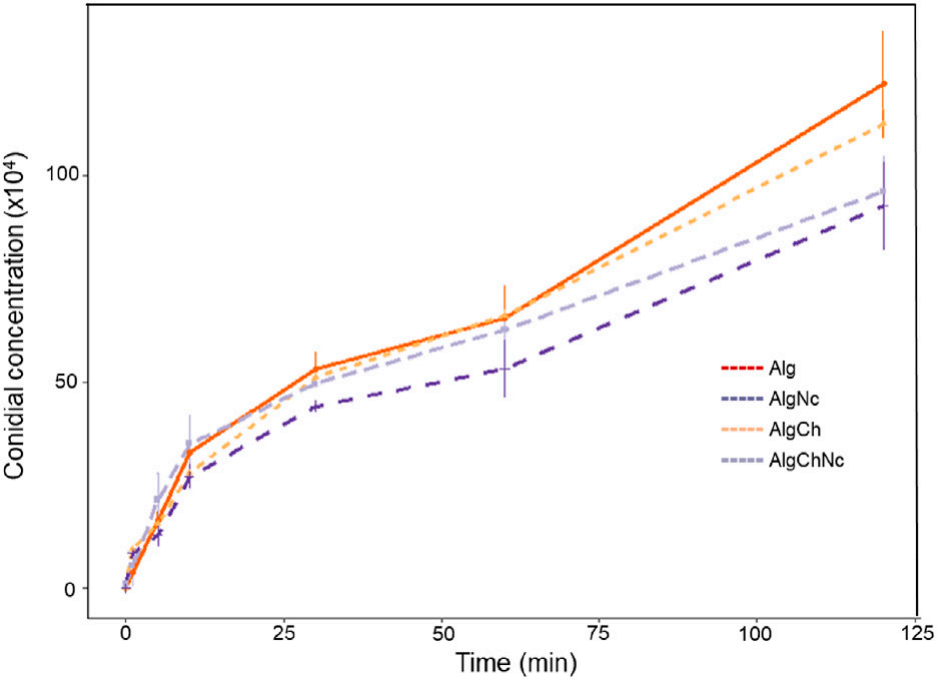
Release rate of encapsulated conidia in the different formulations developed.

The results of the release rate of conidia from the formulated microcapsules are shown in Figure 10. It was determined that the sodium alginate (Alg) microcapsules had the highest release rate, with about 66% of conidia released at 60 min and 100% at 100 min. Microcapsules with sodium alginate and chitosan (AlgCh) had similar behavior at 60 min, however, 100% of conidia were released near 110 min. Otherwise, microcapsules with sodium alginate, chitosan and nanocellulose (AlgChNc) showed a slower release rate, with about 65% of conidia released at 60 min and 93% at 120 min. Finally, microcapsules with sodium alginate and nanocellulose (AlgNc) had the slowest release rate, with 51% of conidia released at 60 min and about 85% at 120 min.

### 3.5 Conidial temperature tolerance

**FIGURE 11:**
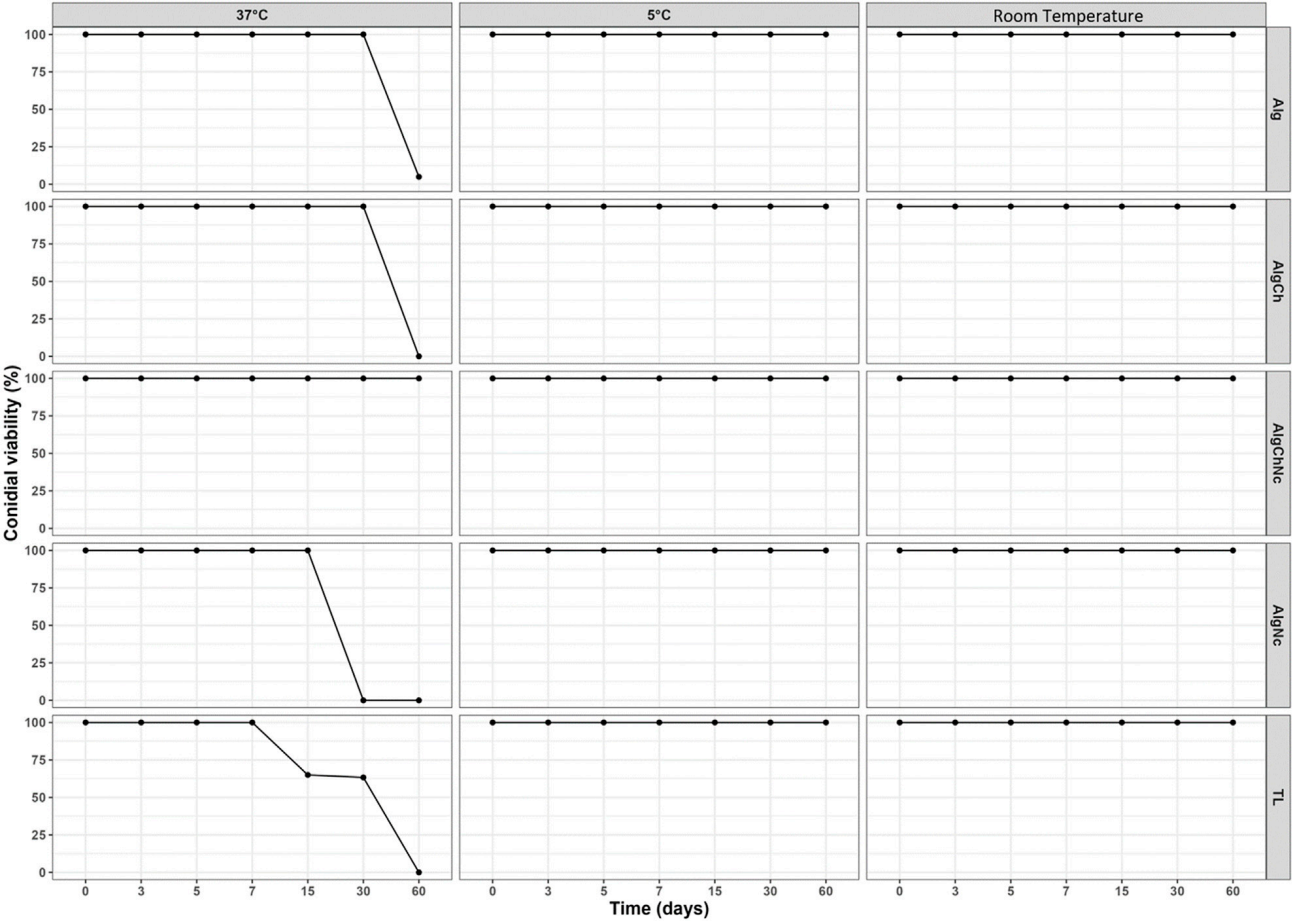
Conidial viability of 4 microencapsulated *T. longibrachiatum* in Alg, AlgCh, AlgChNc and AlgNC, and a conventional formulation (TL), exposed to three different storage temperatures (5, room temperature and 37°C).

The results of the conidial viability of microencapsulated *T. longibrachiatum* exposed to three different storage temperatures (5, room temperature and 37°C) are shown in Figure 11. It was determined that at room temperature (20^o^C) and at 5°C the four microcapsule formulations (Alg, AlgNc, AlgCh and AlgChNc) and conventional formulation (TL) maintained 100% viability for 2 months (Figure 11). When conidial viability was evaluated at a temperature of 37^o^C (80% relative humidity), it was found that the TL formulation had a decrease in conidial viability from day 7 of exposure (65%), which reached 0% at 60 days. On the other hand, Alg and AlgCh microcapsules decreased their conidial viability to 0% at 60 days. The AlgNc microcapsules decreased their conidial viability from 15 days of exposure until reaching 0 at day 30. The best result was obtained with the AlgChNc formulation, which maintained 100% conidial viability during the 60 days of evaluation.

### 3.6 Biological activity - antagonism *in vitro*

**FIGURE 12:**
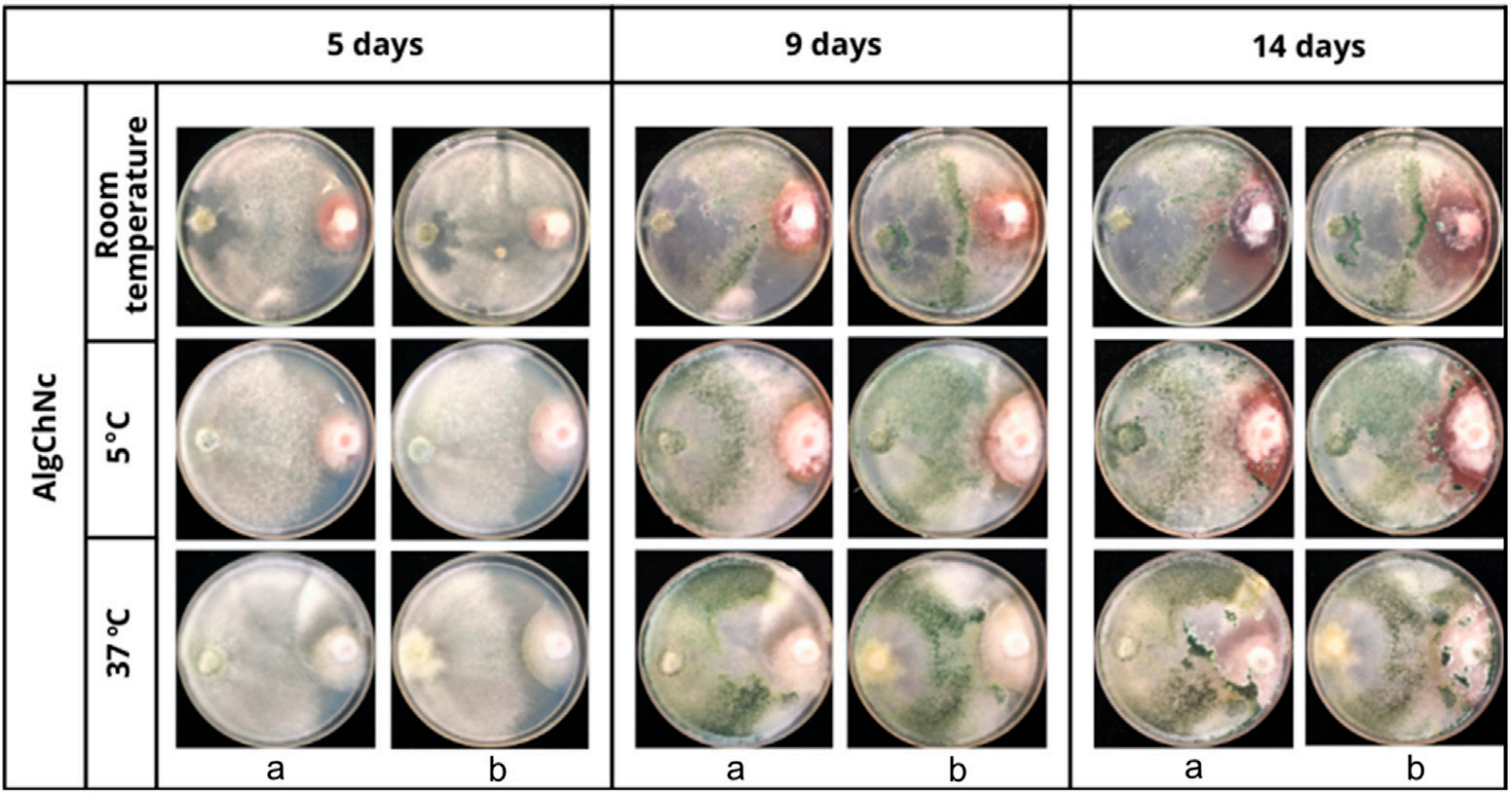
*In vitro* antagonism of AlgChNc + *T. longibrachiatum* **(A)** vs. *F. oxysporum* **(B)** following exposure of the formulation to three different temperatures.

The results of the antagonism tests showed that *T. longibrachiatum* grew over at least two-thirds of the surface of the medium, finding a class 2 degree of antagonism (Figure 12). The four formulations evaluated behaved in the same way and the temperature to which the microcapsules were exposed prior to the antagonism tests did not influence them.

## 4 Discussion

The findings of this research have significant implications for modern agriculture, particularly in the field of sustainable pest management. By providing an effective method for enhancing the stability and viability of *T. longibrachiatum* under various environmental stresses and reducing the dependence of chemical pesticides, this study contributes to the advancement of biopesticide applications. Moreover, by extending the shelf-life and improving the efficacy of biological agents, this research has the potential to transform pest management strategies, especially in regions with challenging environmental conditions.

### 4.1 Standardization of microcapsule formulation

Tests conducted to standardize the formulation of alginate microcapsules using Span 80 and Tween 20 surfactants determined that microcapsules formulated with Span 80 tended to agglomerate with highly heterogeneous sizes. Similarly, Yang et al. (2004) obtained irregularly shaped chitosan microcapsules using Span 80, however, the combination of Span 80 and Tween 60 produced spherical microcapsules with smooth surfaces. In contrast, the microcapsules formulated with Tween 20, presented regular circumference, and smooth surface, which agrees with what has been reported by other authors (Hwang et al., 2006; Martins et al., 2017). In addition, this formulation presented microcapsules more homogeneous and with no tendency to agglomeration. Similarly, Martins et al. (2017) found that the addition of Tween 20 (0.5% and 1.0% v/v) resulted in perfectly spherical microcapsules, because the surfactant reduced the surface tension of the alginate solution and thus the interfacial tension between the emulsion droplet and the alginate solution, which accelerated droplet detachment and the production of spherical capsules. This may be associated with the more condensed molecular structure of Tween 20, which allows for faster diffusion and reorganization on the droplet surface (Silva et al., 2019).

With respect to the effects of stirring speed on the morphology of the microparticles, it was determined that the higher speed, 1,000 rpm, decreased the size and increased the homogeneity. These results agree with the findings of other researchers, who determined that an increase in homogeneity and smaller size of the microparticles formed, could be due to the fact that a higher agitation speed provides a higher shear force, which allows better dispersing the droplets and avoiding agglomeration of the microcapsules (Li et al., 2021; Ma et al., 2020; Shi et al., 2019). Ma et al. (2020) obtained smaller size microcapsules by increasing the stirring speed to 1,000 rpm, like what was observed in this research. On the other hand, Shi et al. (2019) evaluated different agitation speeds (600, 800, 1,000, and 1,200 rpm) and obtained spherical and uniform nanocapsules, however, at 1,000 rpm they had a more homogeneous size.

Regarding the concentration of the surfactant Tween 20 (5%, 7.5%, and 10%), and in agreement with our results, other authors have determined that a higher concentration of Tween 20 increases the sizes of microcapsules (Teo et al., 2016). This could be because the increase in emulsion droplet size with increasing surfactant concentration may result in higher surfactant viscosity at the interface, preventing fluidity and easy movement of the organic phase into the aqueous phase (Barzegar et al., 2018). According to Baranauskaite et al. (2019) found that the optimal composition for their microcapsule formulation included the addition of Tween 20 at 4.7%, which is consistent with the percentage determined in this study.

### 4.2 Nanocellulose synthesis

Synthesis of nanocellulose from cotton cellulose using a sonication method, determined that this protocol is adequate. Sonication is a strategy based on an ultrasound mechanism that causes natural fibers to break down into nanofibers in water (Zhao et al., 2007), this method allows breaking micrometer aggregates and stabilizing suspensions (Gouveia et al., 2020). Soni and Mahmoud (2015) found that following treatment with ultrasonication, cotton stalk fibers were reduced to sizes of approximately 20 nm in length and 100–500 nm in diameter. When sonication is applied at a high intensity level, such as that used in this research (700 W), it results in the disruption of the strong glycosidic bonds of the crystalline portion of cellulose, which, together with the disruption of the interconnected network of hydrogen bridges, leads to the opening or defibrillation of the structure (Abd et al., 2016).

### 4.3 Characterization of microcapsules

#### 4.3.1 Scanning electron microscopy (SEM)

The size of the alginate microcapsules decreased with the incorporation of the conidia, in accordance with the findings of De Oliveira et al. (2020). On the other hand, the change from a smooth to a rough surface, in the alginate and nanocellulose formulation, after encapsulation has been reported by Rungraung et al. (2022), who mention that the addition of nanocellulose to alginate microcapsules generates rough surfaces with irregularities. This roughness may be related to the drying process of the microcapsules and to the strong ionic crosslinking interactions (Brondi et al., 2022). In the case of the alginate and chitosan formulation, the roughness or scratched and fibrous surfaces may be due to the presence of chitosan on the microcapsule surface (Jurić et al., 2019). Moreover, the microcapsules formulated with chitosan, alginate and nanocellulose, presented regular circumference and roughness, and similar to what happened with the other formulations and after encapsulation they tended to agglomerate and show rough surfaces. Jurić et al. (2021) mention that agglomeration may be due to the size of the microcapsules and the presence of water molecules causing them to bind together upon drying. The rough surfaces observed on microcapsules with conidia may be since as conidia are introduced into the polymeric system, the uniform distribution of conidia throughout the matrix may make it possible to notice a roughness (Batista et al., 2017). Jurić et al. (2019) found that microcapsules prepared with *T. viride* conidia had numerous oval dimples and a germ tube was observed to cross the surface of the microcapsule within a few days. Near-surface sections revealed the presence of hyphae and mycelium formation within the matrix, which may account for the surface characteristics of microcapsules containing conidia, as the presence of *Trichoderma* and mycelial growth within the microcapsule change the structure of the gel network. It has been found that the drying process of microcapsules at room temperature causes a loss of sphericity and the surface becomes rough with irregular wrinkles, which is related to the relaxation of biopolymer tension due to water and moisture loss (Jurić et al., 2019; Rodrigues et al., 2020).

#### 4.3.2 Thermogravimetric analysis (TGA)

The addition of chitosan to microcapsule formulations with alginate has been found to improve physicochemical properties, with enhanced stability in swelling media of different pH and improved structural strength (Qu and Luo, 2020). Hashim et al. (2019) mention that chitosan improves hydrogel stability due to the formation of polyelectrolyte complexes between the positively charged amino groups and the carboxylic residues of alginate. Siqueira et al. (2019) have determined that alginate formulations present higher stability when they contain nanocellulose in their composition. For the temperature range to which the commercial formulations could be exposed, it can be observed how the addition of polymers such as nanocellulose and chitosan, provide greater protection to the conidia, since the incorporation of both increases the stability to temperature degradation, decreasing the weight loss compared to the Alg formulation.

#### 4.3.3 Differential scanning calorimetry (DSC)

The peak at 75°C observed in the Alg, AlgNc, and AlgChNc microcapsules, is attributed to the loss of water from the hydrophilic groups of alginate (Lim and Ahmad, 2017) and the moisture loss from nanocellulose (Mohamed et al., 2021). Additionally, the AlgCh formulation exhibits a peak near 80°C, which is similarly associated with the evaporation of absorbed water (Nikoo et al., 2018).

#### 4.3.4 Fourier transform infrared spectroscopy (FTIR)

The FTIR spectra of the microcapsule formulations without conidia and with conidia, respectively. It can be noted that both spectra are very similar. A band at 3,357 cm^−1^ was observed. In addition, two wave peaks are observed at 1,595 cm^−1^ and 1,417 cm^−1^. The peak observed at 2,925 cm^−1^ refers to the C-H bending vibration in the methylene group (Nandiyanto et al., 2023). Meanwhile, the stretching of the N-H bond with the C=O (amide) group observed in the 1,622 cm^−1^ peak of sodium alginate and chitosan microcapsules suggests an interaction between the carboxyl and amino group of the polymers (Bulatao et al., 2017). The disappearance of the amino band of chitosan at 1,592 cm^−1^ (Table 1) may suggest the formation of a polyelectrolyte complex between sodium alginate and chitosan, furthermore, the new peak observed at 1,739 cm^−1^, belonging to the COOH group, is an indication of the acidic condition in which the microcapsules were prepared (Khorshidian et al., 2024). The sodium alginate, chitosan and nanocellulose (AlgChNc) formulation showed similar bands to the AlgCh formulation.

### 4.4 Encapsulation efficiency

A high encapsulation efficiency of more than 92% was found in all formulations. These encapsulation percentages are higher than those reported by other authors (Liu and Liu, 2009; Mancera et al., 2019). For example, Mancera et al. (2019) obtained an encapsulation efficiency of 80% in the formulation of calcium alginate microcapsules using the emulsion-internal gelation method. Meanwhile, Liu and Liu (2009) found that the sodium alginate formulation without any coating had the lowest encapsulation efficiency (45%). This indicates that the microcapsules developed in this research are a suitable method for encapsulation of *T. longibrachiatum* conidia. This high encapsulation efficiency could be related to that in the emulsion-internal gelation method, as CaCO_3_ is finely dispersed in the alginate solution, Ca^2+^ dissociated by acetic acid can gel *in situ* without disrupting the emulsion droplet to maintain the spherical shape (Huang et al., 2021).

### 4.5 Conidial release rate

The slower conidial release rate of the AlgCh in comparison to Alg microcapsules, have been reported previously. Belščak et al. (2015) found that caffeine release was slower when alginate microcapsules were reinforced with chitosan, this because this matrix was the least susceptible to rehydration, as the rate of water diffusion through the active ingredient coating is the limiting step for the rate of expansion and release. Otherwise, formulations containing nanocellulose had the slowest release of conidia, which may be attributed to the polysaccharide’s ability to restrict the movement of alginate chains (Lin et al., 2011), or high physical entanglement (Supramaniam et al., 2018) slowing release. Faster release of total conidia has been observed in another research (Liu and Liu, 2009; Aziz et al., 2015). Microcapsules that provide a more controlled release may require fewer applications, reducing costs and improving microbial viability (Adzmi et al., 2021). Cells that are gradually released from microcapsules, by degradation of the polymeric matrix, are more protected against destructive factors than uncoated microorganisms (Szczech and Maciorowski, 2016).

The results indicate that, with increasing time, the amount of released conidia increases, observing a positive correlation, like that reported by other authors (Adzmi et al., 2021; Vinceković et al., 2016). This could be due to various factors such as microcapsule surface moisture, water penetration into the microcapsule, transition from glassy to rubbery phase of polymers, swelling, diffusion of charged agents through the microcapsule matrix and surface layer, desorption from the surface, disintegration, dissolution, or erosion of the microcapsule structure (Vinceković et al., 2017).

In microencapsulation, the release of encapsulated cells is a crucial indicator of the sustained release effect and durability of the microcapsules (Qi et al., 2023). The cells can survive, and their metabolic activity can be maintained for extended periods, with controlled release occurring as they adapt to the surrounding environmental conditions. Encapsulated microorganisms can be significantly more efficient than conventional powder and liquid formulations (John et al., 2011). Vinceković et al. (2017) state that optimal release can be achieved through continuous release over an extended period, providing prolonged protection and nutritional effects for plants. It is important to note that the release rates observed in this study were determined under simulated *in vitro* conditions. However, under real field conditions, these results may vary, as several factors can influence release, such as climatic conditions, including temperature, humidity, rainfall, solar radiation, wind, and the presence of other microorganisms. For this reason, subsequent studies should be conducted under various real field conditions.

### 4.6 Conidial temperature tolerance

The AlgChNc formulation yielded the best results, maintaining 100% conidial viability throughout the entire 60-day evaluation period. This indicates that the combination of polymers such as alginate, chitosan and nanocellulose for the formulation of microcapsules allows the protection of *T. longibrachiatum* conidia, when exposed to temperatures between 5°C and 37°C. The maintenance of viability observed in AlgChNc microcapsules could be related to the fact that the combination of polymer matrices can retard oxygen diffusion in the microcapsules, which limits the amount of oxygen available to participate in the oxidation of macromolecules, as well as cause oxidative stress (Ishak et al., 2021; Cruz et al., 2022). The protection given by microcapsules leads to longer shelf life and maintenance of metabolic activity for long periods of time, both during storage and after application. On the other hand, higher temperatures can lead to greater reactivity and diffusion of reactive oxygen species (ROS), decreasing the viability of conidia (Ishak et al., 2021; Braga et al., 2022).

The maintenance of viability of encapsulated conidia in AlgChNc microcapsules is a promising result for commercial use and contrasts with the findings of other researchers who have determined that microencapsulated conidia maintain their viability at low storage temperatures (5°C–8°C), but that their viability decreases as temperature increases. For example, Przyklenk et al., 2017, determined that microencapsulated conidia of *Metarhizium sp.* remain viable at storage temperatures of 5°C for a period of 6 months, however, viability decreased at 25°C. De Oliveira et al. (2020) evaluated the exposure of microencapsulated *Trichoderma* conidia at 8^o^C, 25^o^C, and 35^o^C, finding that the best viability maintenance was obtained for lyophilized microcapsules stored at 8^o^C, however, there was decrease in viability at a temperature of 25^o^C. Adzmi et al. (2021) found that the best maintenance of viability of microencapsulated *Trichoderma* conidia was obtained at a temperature of 5^o^C. On the other hand, Szczech and Maciorowski (2016) managed to maintain the viability of *Trichoderma* conidia almost at the same level (10^4,5^ cfu.g^−1^) in freeze-dried microcapsules, after 6 months of storage at 4^o^C, however the viability of conidia decreased in wet microcapsules, at storage temperatures of 30°C after 15 days, and after 90 days the spores were no longer viable.

### 4.7 Biological activity - antagonism *in vitro*

*T. longibrachiatum* showed a class 2 degree of antagonism by growing over two-thirds of the medium, and the four formulations evaluated exhibited consistent behavior without influence from the prior temperature exposure. Several investigations have determined that some species of the genus *Trichoderma* have a high antagonistic capacity with inhibition percentages of 50.68% (Redda et al., 2018), and 92.11% (Nofal et al., 2021) in the *in vitro* control of *F. oxysporum*, which coincides with what was observed in this research and demonstrates the conservation of the antagonistic capacity of the fungus after microencapsulation. Sundaramoorthy and Balabaskar (2013) obtained a growth inhibition of 27.22% when they used *T. longibrachiatum* for *in vitro* control of *F. oxysporum*. Sallam et al. (2019) on their part found that *T. longibrachiatum* reduced the severity of wilt disease by 24.8%.

Furthermore, it is important to highlight both the cost and effectiveness of the microencapsulation method. The cost of encapsulation depends on various factors, including production scale, the type and quantity of additives used, and the specific protocols employed, all of which influence energy consumption and the use of laboratory equipment. However, in this research, the protocol used involves simple equipment, as an ultra-turrax is employed for the generation of the microcapsules, in contrast to other protocols described in the literature. The use of natural biopolymers such as alginate, chitosan, and nanocellulose offers a cost-effective and environmentally sustainable alternative to synthetic polymers. Additionally, this approach enhances the shelf life and stability of *Trichoderma* conidia, providing a dual benefit: it ensures an eco-friendly solution while extending the efficacy and availability of these biocontrol agents over prolonged periods.

## 5 Conclusion

The results of this research demonstrate that the polymers sodium alginate, chitosan and nanocellulose allow the formulation of microcapsules that help to protect and maintain the viability of *T. longibrachiatum* conidia under different temperature conditions. An encapsulation efficiency above 92% was obtained for the three microcapsule formulations and the viability of the conidia was maintained when the microcapsules were stored at 5^o^C, 20^o^C, and 37^o^C, being the formulation with the three polymers (AlgChNc) the one that showed the best result when stored for 2 months at the temperature of 37°C. *In vitro* antagonism tests showed that microencapsulation of the fungus does not affect its antagonistic capacity, obtaining a category II in substrate competition against the phytopathogenic fungus *F. oxysporum for the* three microcapsule formulations; however, tests are required to determine whether this capacity is maintained under field conditions.

## Data Availability

The original contributions presented in the study are included in the article/Supplementary Material, further inquiries can be directed to the corresponding author.
