# Corrigendum: Evaluation of the viability of microencapsulated *Trichoderma longibrachiatum* conidia as a strategy to prolong the shelf life of the fungus as a biological control agent

**DOI:** 10.3389/fchem.2025.1562696

**Published:** 2025-02-17

**Authors:** Luis Diego Arias-Chavarría, Diego Batista-Menezes, Steffany Orozco-Cayasso, Alejandro Vargas-Martínez, José Roberto Vega-Baudrit, Gabriela Montes de Oca-Vásquez

**Affiliations:** ^1^ Escuela de Ciencias Agrarias, Universidad Nacional, Heredia, Costa Rica; ^2^ National Nanotechnology Laboratory, National Center for High Technology, San José, Costa Rica; ^3^ Laboratorio de Fitopatología, Escuela de Ciencias Agrarias, Universidad Nacional, Heredia, Costa Rica; ^4^ Center for Sustainable Development Studies, Universidad Técnica Nacional, Alajuela, Costa Rica

**Keywords:** microcapsules, alginate, nanocellulose, chitosan, phytopathogenic controller

In the published article, there was an error in the affiliation numbers of the author **Gabriela Montes de Oca-Vásquez**. The correct affiliations appear above.

The authors apologize for this error and state that this does not change the scientific conclusions of the article in any way. The original article has been updated.

