# Supplementary material for: Evaluation of the viability of microencapsulated Trichoderma longibrachiatum conidia as a strategy to prolong the shelf life of the fungus as a biological control agent
Source: Front Chem. 2025 Jan 15;12:1473217. doi: 10.3389/fchem.2024.1473217 (PMC11775737; doi:10.3389/fchem.2024.1473217)
Supplement: Supplementary file 1 [file Table1.docx]

**Supplementary material**

**Supplementary table 1.** Absorption bands of the compounds used for the different microcapsule formulations.

| **Compound** | **Wavenumber (cm^-1^)** | **Functional group** | **Reference** |
| --- | --- | --- | --- |
| **Sodium alginate** | 1028 | C-O-C | Derkach et al. 2019. |
|  | 1409 | COO- | Li et al. 2016. |
|  | 1595 | COO- | Li et al. 2016. |
|  | 3257 | O-H | Essifi et al. 2021. |
| **Chitosan** | 1025 | C-O-C | Mu et al. 2022. |
|  | 1152 | C-O-C | Khorshidian et al. 2019. |
|  | 1374 | N-H (Amide III) | Khorshidian et al. 2019. |
|  | 1592 | N-H (Amide II) | Vaziri et al. 2018. |
|  | 1651 | C=O (Amide I) | Vaziri et al. 2018. |
|  | 2867 | C-H | Khorshidian et al. 2019. |
|  | 3358 | -NH2 and -OH | Huo et al. 2018. |
| **Nanocellulose** | 1017 | C-O-C | Jordan et al. 2021. |
|  | 1372 | C-O | Pandi et al. 2021. |
|  | 1652 | O-H | Li et al. 2021. |
|  | 3359 | O-H | Theivasanthi et al. 2018. |
| **Calcium carbonate** | 712 | CO_3_ (out-of-plane bend) | Al-Hosney and Grassian, 2005. |
|  | 874 | CO_3_ (asymmetric stretch) | Al-Hosney and Grassian, 2005. |
|  | 1416 | C-O (in-plane bend) | Böke et al. 2004. |
|  | 1794 | Combination band | Al-Hosney and Grassian, 2005. |
|  | 2509 | Combination band | Al-Hosney and Grassian, 2005. |
| **Calcium chloride** | 1627 | H-O-H | Araujo et al. 2021 |
|  | 3393 | -OH | Jurić et al. 2019. |

**References**

Al-Hosney, H., Grassian V. (2005). Water, sulfur dioxide and nitric acid adsorption on calcium carbonate: A transmission and ATR-FTIR study. Physical Chemistry Chemical Physics, 7(6), 1266-1276. doi: [10.1039/B417872F](https://doi.org/10.1039/B417872F)

Araujo, J. A., Cortese, Y. J., Mojicevic, M., Brennan Fournet, M., & Chen, Y. (2021). Composite films of thermoplastic starch and CaCl2 extracted from eggshells for extending food shelf-life. *Polysaccharides*, 2(3), 677-690. doi: 10.3390/polysaccharides2030047

Böke, H., Akkurt, S., Özdemir, S., Göktürk, E., Saltik, E. (2004). Quantification of CaCO3–CaSO3· 0.5 H2O–CaSO4· 2H2O mixtures by FTIR analysis and its ANN model. Materials Letters, 58(5), 723-726. doi: [10.1016/j.matlet.2003.07.008](https://doi.org/10.1016/j.matlet.2003.07.008)

Derkach, S.R., Voron’ko, N.G., Sokolan, N.I., Kolotova, D.S., Kuchina, Y.A. (2019). Interactions between gelatin and sodium alginate: UV and FTIR studies. Journal of Dispersion Science and Technology, 41(5), 690-698. doi: [10.1080/01932691.2019.1611437](http://dx.doi.org/10.1080/01932691.2019.1611437)

Essifi, K., Lakrat, M., Berraaouan, D., Fauconnier, M., El-Bachiri, A., Tahani, A. (2021). Optimization of gallic acid encapsulation in calcium alginate microbeads using Box-Behnken Experimental Design. Polymer Bulletin, 78(10), 5789-5814. doi: [10.1007/s00289-020-03397-9](https://link.springer.com/article/10.1007/s00289-020-03397-9).

Huo, X., Li, W., Wang, Y., Han, N., Wang, J., Wang, N., Zhang, X. (2018). Chitosan composite microencapsulated comb-like polymeric phase change material via coacervation microencapsulation. Carbohydrate polymers, 200, 602-610. doi: [10.1016/j.carbpol.2018.08.003](https://doi.org/10.1016/j.carbpol.2018.08.003)

Jordan, J., Cheng, H., Easson, M., Yao, W., Condon, B., Gibb, B. (2021). Effect of Nanocellulose on the Properties of Cottonseed Protein Isolate as a Paper Strength Agent. Materials, 14(15), 4128. doi: [10.3390/ma14154128](https://doi.org/10.3390%2Fma14154128)

Jurić, S., Đermić, E., Topolovec-Pintarić, S., Bedek, M., Vinceković, M. (2019). Physicochemical properties and release characteristics of calcium alginate microspheres loaded with Trichoderma viride spores. Journal of Integrative Agriculture, 18(11), 2534-2548. doi: [10.1016/S2095-3119(19)62634-1](https://doi.org/10.1016/S2095-3119(19)62634-1)

Khorshidian, N., Mahboubi, A., Kalantari, N., Hosseini, H., Yousefi, M., Arab, M., Mahdavi, F. (2019). Chitosan-coated alginate microcapsules loaded with herbal galactagogue extract: Formulation optimization and characterization. Iranian Journal of Pharmaceutical Research, 18(3), 1180. doi: [10.22037/ijpr.2019.1100776](https://doi.org/10.22037/ijpr.2019.1100776)

Li, J., Kim, S., Chen, X., Park, H. (2016). Calcium-alginate beads loaded with gallic acid: Preparation and characterization. LWT - Food Science and Technology, 68, 667–673. doi: [10.1016/j.lwt.2016.01.012](http://dx.doi.org/10.1016/j.lwt.2016.01.012)

Li, M., He, B., Chen, Y., Zhao, L. (2021). Physicochemical properties of nanocellulose isolated from cotton stalk waste. ACS Omega, 6(39), 25162-25169. doi: [10.1021/acsomega.1c02568](https://doi.org/10.1021/acsomega.1c02568)

Mu, H., Song, Z., Wang, X., Wang, D., Zheng, X., Li, X. (2022). Microencapsulation of algae oil by complex coacervation of chitosan and modified starch: Characterization and oxidative stability. International Journal of Biological Macromolecules, 194, 66-73. doi: [10.1016/j.ijbiomac.2021.11.168](https://doi.org/10.1016/j.ijbiomac.2021.11.168)

Pandi, N., Sonawane, S., Kishore, K. (2021). Synthesis of cellulose nanocrystals (CNCs) from cotton using ultrasound-assisted acid hydrolysis. Ultrasonics sonochemistry, 70, 105353. doi: [10.1016/j.ultsonch.2020.105353](https://doi.org/10.1016/j.ultsonch.2020.105353)

Theivasanthi, T., Christma, F.A., Toyin, A.J., Gopinath, S.C., Ravichandran, R. (2018). Synthesis and characterization of cotton fiber-based nanocellulose. International journal of biological macromolecules, 109, 832-836. doi: [10.1016/j.ijbiomac.2017.11.054](https://doi.org/10.1016/j.ijbiomac.2017.11.054)

Vaziri, A., Alemzadeh, I., Vossoughi, M. (2018). Improving survivability of Lactobacillus plantarum in alginate-chitosan beads reinforced by Na-tripolyphosphate dual cross-linking. LWT, 97, 440-447. doi: [10.1016/j.lwt.2018.07.037](https://doi.org/10.1016/j.lwt.2018.07.037)
