# Supplementary material for: Evaluation of the viability of microencapsulated Trichoderma longibrachiatum conidia as a strategy to prolong the shelf life of the fungus as a biological control agent
Source: Front Chem. 2025 Jan 15;12:1473217. doi: 10.3389/fchem.2024.1473217 (PMC11775737; doi:10.3389/fchem.2024.1473217)

**Supplementary material**

**Supplementary table 2.** *In vitro* antagonism of microencapsulated *T. longibrachiatum* conidia vs *F. oxysporum* following exposure of the formulation to three different temperatures


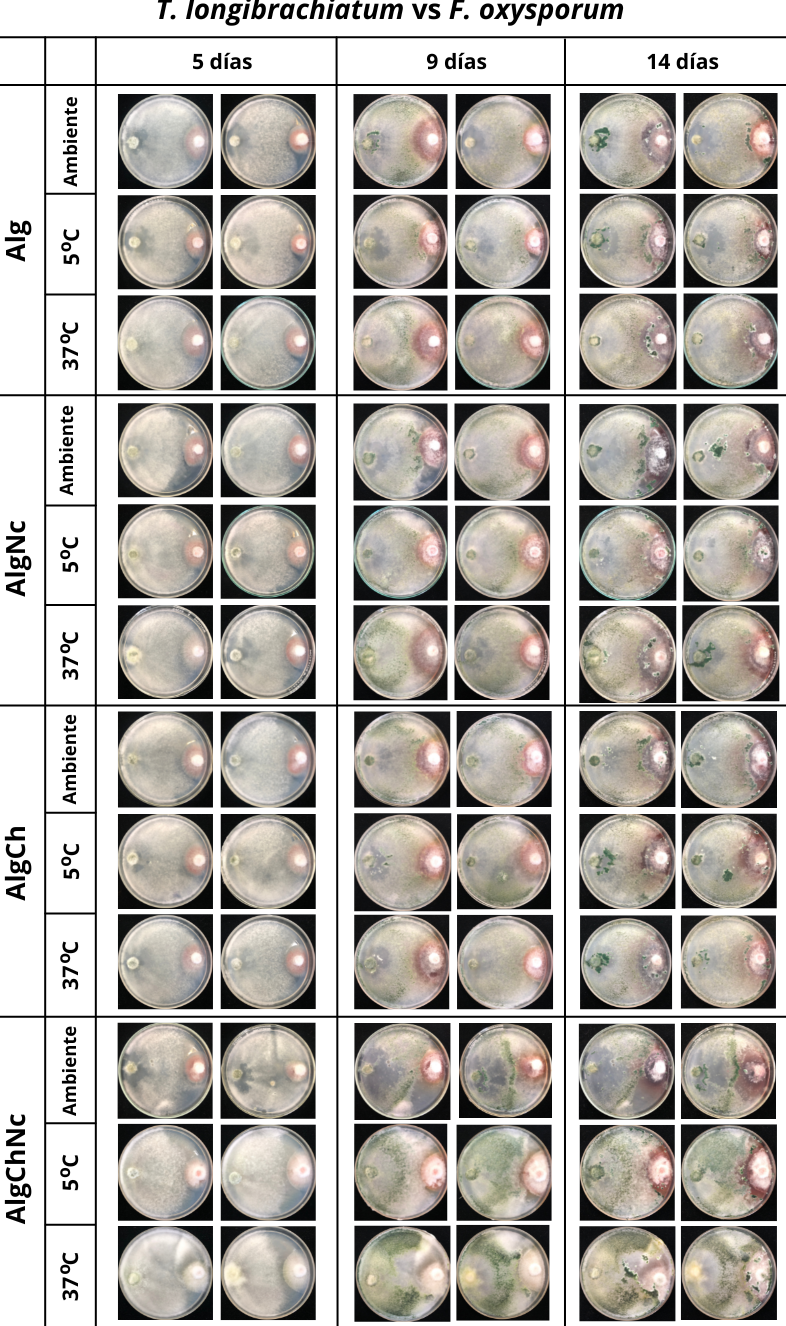

Supplement: Supplementary file 2 [file Table2.docx]
